# A Case of Posterior Polar Hemispheric Choroidal Dystrophy Successfully Diagnosed With Ultra-Widefield Fundus Autofluorescence and Optical Coherence Tomography Angiography

**DOI:** 10.7759/cureus.55878

**Published:** 2024-03-10

**Authors:** Chinatsu Takano, Shuntaro Ogura, Hironori Ozeki, Tsutomu Yasukawa, Miho Nozaki

**Affiliations:** 1 Department of Ophthalmology and Visual Science, Nagoya City University Graduate School of Medical Sciences, Nagoya, JPN; 2 Ophthalmology, Ozeki Eye Clinic, Kanie, JPN; 3 Department of Ophthalmology, Laser Eye Center, Nagoya City University East Medical Center, Nagoya, JPN

**Keywords:** hypofluorescence, retinal pigment epithelium, optical coherence tomography angiography, ultra-wide field fundus autofluorescence, hemispheric peripapillary chorioretinal atrophy

## Abstract

We report a case of a 78-year-old man presenting with uncertain visual field loss, ultimately identified as posterior polar hemispheric choroidal dystrophy (PPHCD) using ultra-widefield fundus autofluorescence (FAF) and optical coherence tomography angiography (OCTA). The patient initially reported blurred vision in the left eye and had a previous diagnosis of suspected bilateral normal tension glaucoma based on optic nerve head excavation and static perimetry measurements. Detailed examination revealed suspicious retinal atrophy. Notably, the patient had a tigroid fundus, which complicated the correlation between visual field defect and chorioretinal atrophy. Ultra-widefield FAF highlighted mosaic/patchy hypofluorescent areas, emphasizing this atrophy. OCTA images confirmed choriocapillaris loss in the hemispheric choroidal atrophy and parafoveal atrophy. The combination of these imaging techniques enabled a definitive diagnosis of PPHCD. Long-term follow-up and continued investigation with these imaging modalities may hold promise for a better understanding of disease progression and management in similar cases.

## Introduction

Posterior polar hemispheric choroidal dystrophy (PPHCD) was initially described in 2010 as a fundus abnormality characterized by annular and hemispherical retinal and choroidal atrophy while sparing the fovea [1]. However, due to its rarity, only a limited number of cases have been reported so far [2,3]. Here, we present a case of PPHCD that was successfully diagnosed using ultra-widefield fundus autofluorescence (FAF) images and optical coherence tomography angiography (OCTA), as conventional fundus examination alone posed challenges in diagnosing this condition.

## Case presentation

**Figure 1:**
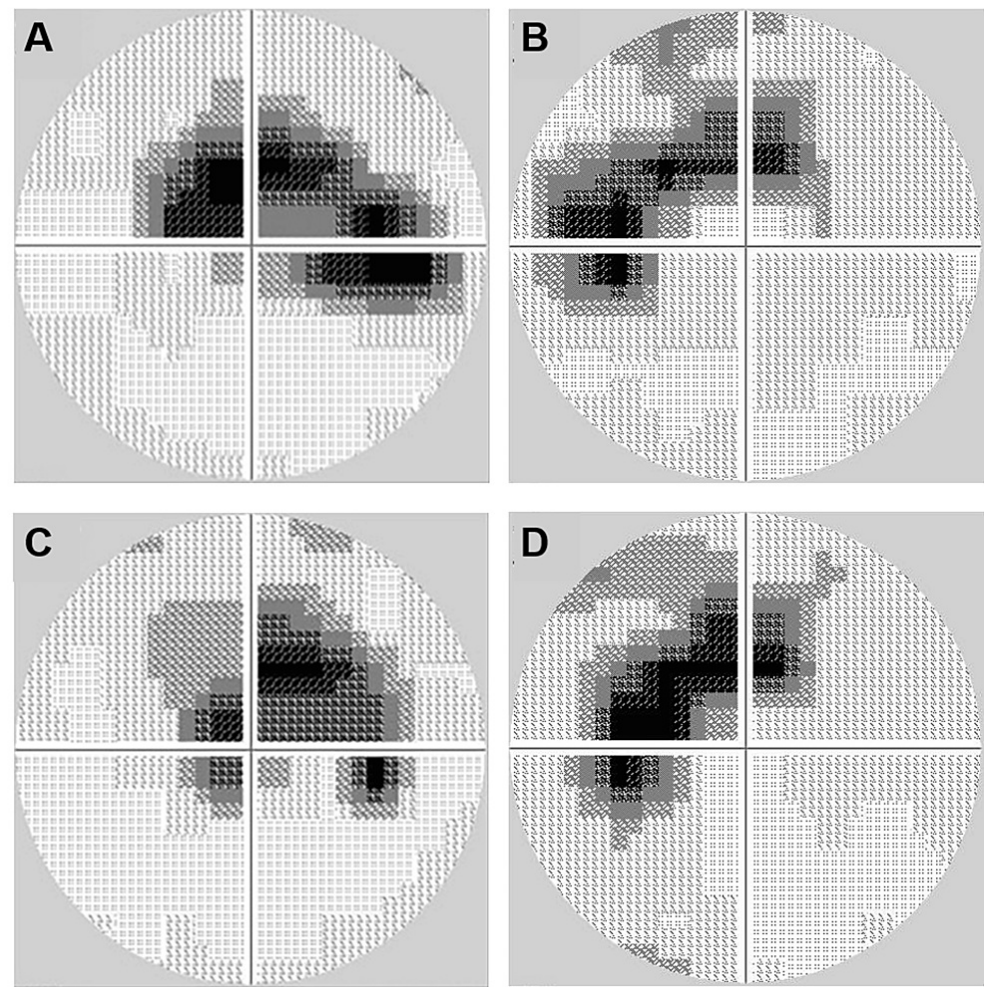
Scotoma in visual field measurements A-B: Visual fields were measured using Humphrey perimetry (30-2 program). C-D: No progression of scotoma was observed after one year.

A 78-year-old male patient with left eye blurred vision was diagnosed with cataracts and subsequently underwent bilateral cataract surgery. He had a past medical history that included IgG4-related lung disease, hypertension, asthma, and atrial fibrillation. All of these conditions were well-controlled with the following medications: bisoprolol fumarate, azilsartan, fluticasone propionate/formoterol fumarate hydrate, and amlodipine besylate. During follow-up at a local ophthalmology clinic, he was prescribed latanoprost eyedrops for suspected normal tension glaucoma due to optic nerve head cup-to-disk ratio enlargement and visual field defects revealed by Humphrey perimetry (Figure 1A-B). There was no progression of scotoma in either eye after a one-year follow-up (Figure 1C-D).

During his follow-up, suspected chorioretinal atrophy in his fundus raised dystrophy concerns. While night blindness was not evident, the patient reported ongoing issues with dark adaptation. Additionally, his family noticed difficulty recognizing faces, leading to a referral for comprehensive assessment and diagnosis at our hospital.

**Figure 2:**
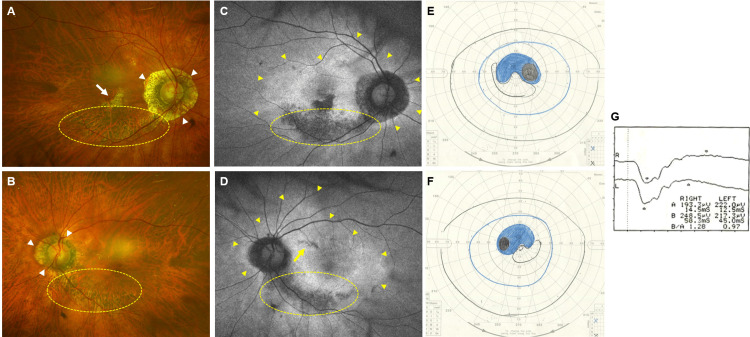
Interrelation of fundus findings, visual field, and electroretinogram A-B: Fundus photographs revealed bilateral peripapillary atrophy (white arrowheads), more dominant in the right eye, and choroidal atrophy extending from the optic disk to the lower temporal peripapillary region (yellow-dotted oval). Due to the tigroid fundus appearance, chorioretinal atrophy was unclear in the fundus photography. C-D: In contrast, FAF showed well-defined hypofluorescent areas corresponding to the atrophy. Additionally, annular hyperfluorescent areas were detected with FAF in both eyes (yellow arrowheads). A definite chorioretinal atrophy in the parafovea was also observed in the right eye (see white arrow in image A). Isolated hypofluorescence, not clearly visible in the fundus photography, was detectable with FAF (yellow arrow). E-F: Visual fields measured using Goldmann perimetry exhibited bilateral symmetrical scotomas corresponding to the hypofluorescence areas seen in FAF. G: Full-field electroretinography showed no significant abnormalities. FAF - fundus autofluorescence

The best corrected visual acuity was 20/25 in both eyes. Intraocular pressure was recorded as 10 mmHg in the right eye and 11 mmHg in the left eye. Axial lengths measured by IOLMaster 500 (Carl Zeiss Meditec, Dublin, CA, USA) were 23.5 mm and 23.3 mm in the right and left eyes, respectively. The patient's fundus exhibited a tigroid appearance, characterized by a tessellated pattern and heightened visibility of choroidal vasculature. Bilateral lower hemispheric peripapillary chorioretinal atrophy without vessel constriction was confirmed. Parafoveal atrophy was also observed in the right eye. The cup-to-disk ratio was 0.7 in both eyes, with more pronounced peripapillary atrophy in the right eye (Figures 2A-B). Although fundus examination was hindered by the tigroid appearance, ultra-widefield FAF using a scanning laser ophthalmoscope (Optos, Dunfermline, Scotland) revealed hypofluorescent patches corresponding to the atrophy (Figures 2C-D). These patches aligned with the scotoma seen in Goldmann perimetry (Figures 2-F). Full-field electroretinography (LE-4000, Tomey, Nagoya, Japan) yielded normal results (Figure 2G).

**Figure 3:**
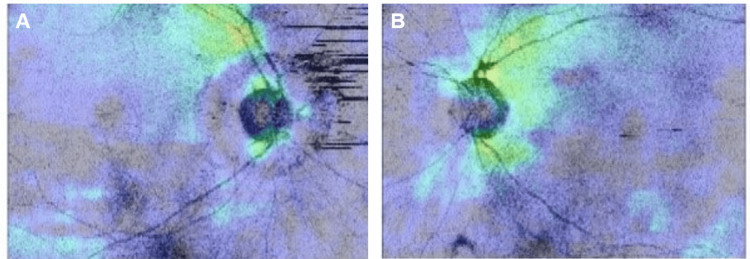
RNFL thinning A comprehensive analysis of the RNFL thickness map in OCT revealed a consistent and general reduction in RNFL thickness observed in both the right (A) and left (B) eyes. RNFL - reduction in retinal nerve fiber layer; OCT - optical coherence tomography

Optical coherence tomography (OCT) analysis (DRI OCT Triton, Topcon, Tokyo, Japan) indicated a general reduction in retinal nerve fiber layer (RNFL) thickness for both eyes (Figure 3).

**Figure 4:**
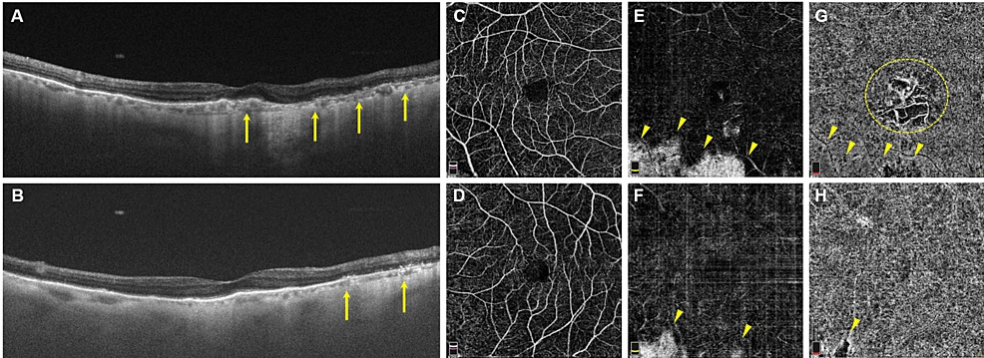
Chorioretinal atrophy visualization through OCT and OCTA A-B: OCT images displayed bilateral choroidal thinning, outer retinal layer atrophy, and RPE loss in FAF hypofluorescent areas (arrows). C-D: OCTA showed normal capillary plexus in the inner retina in both eyes. E-F: Outer retina dropouts were observed as whitened regions (arrowheads). G-H: Choriocapillaris is absent within affected areas (arrowheads). Parafoveal choriocapillaris loss in the right eye enabled a clear view of large choroidal vessels (dotted circle). OCT - optical coherence tomography; OCTA - optical coherence tomography angiography; FAF - fundus autofluorescence; RPE - retinal pigment epithelium

Swept-source OCT imaging demonstrated a noticeable reduction in choroidal thickness and the absence of the outer photoreceptor segment and retinal pigment epithelium (RPE) within the hypofluorescent area observed in FAF. The average choroidal thickness in the normal area was 55.6±8.4 µm, while in the atrophic area, it was 31.0±10.3 µm (p<0.01) in the right eye. In the left eye, the corresponding values were 88.2±27.8 µm and 27.8±15.6 µm (p<0.01), respectively. These measurements are based on the average of five areas adjacent to the upper and lower arcade vessels and are expressed as mean±SD (Figures 4A-B). OCTA (RTVue XR Avanti, Optovue, Fremont, CA, USA) identified outer retina and choriocapillaris loss within hemispheric choroidal atrophy in both eyes, as well as parafoveal atrophy in his right eye. The parafoveal atrophy enabled clear visualization of large choroidal vessels in the Sattler layer and/or Haller layer (Figures 4C-H). Based on these findings, we diagnosed this patient with PPHCD.

## Discussion

PPHCD, a rare condition characterized by chorioretinal atrophy affecting nearly half of the posterior segment, has been the subject of only a limited number of reports to date [1-5]. This case exhibited asymptomatic bilateral and symmetric hemispheric choroidal atrophy, creating diagnostic hurdles during both conventional fundus examination and ultra-widefield fundus photography due to the tigroid fundus appearance. However, the application of ultra-widefield FAF provided clear visualization of the atrophic hypofluorescent regions, aiding in the accurate diagnosis of PPHCD.

It is important to differentiate PPHCD from other conditions that share similar clinical features, such as glaucoma and retinitis pigmentosa (RP). Glaucoma can manifest with scotomas resembling those in PPHCD, but the absence of fundus atrophic lesions distinguishes the two conditions [6]. OCT can be effective in detecting glaucoma, but in this case, the overall reduction in RNFL thickness was attributed to myopia, making it challenging to rule out the possibility of coexisting glaucoma. RP, particularly the sector RP subtype characterized by RP features confined to specific quadrants, is another consideration [7]. However, our patient did not exhibit the hallmark bone spicule-like pigmentation commonly seen along the inferotemporal arcade, a defining characteristic of RP. While RP often has a genetic basis and various mutations have been identified, the genetic underpinnings of PPHCD remain unclear. In this particular case, genetic testing was not conducted since the initial observation was made using ultra-widefield FAF during follow-up. However, forthcoming investigations may necessitate comprehensive genetic analyses, including large panel next-generation sequencing or whole exome sequencing, to effectively exclude other potential dystrophic conditions.

The hypofluorescence observed on FAF indicates a loss of the RPE and overlying photoreceptors, which is known to be linked to impaired visual function [8]. Conversely, hypofluorescence often indicates metabolically stressed RPE [9,10]. Interestingly, patients with RP often exhibit a hyperfluorescent band around the hypofluorescent lesion, potentially indicating future disease progression [8]. In the current case, a bilateral ring-like annular hyperfluorescent area was observed, which aligns with the theory that PPHCD might be associated with annular dystrophy. This form of dystrophy involves continuous atrophy expansion from the optic nerve head [6]. However, in our case, the hypofluorescent area adjacent to the fovea in the right eye appeared denser and was not connected to the sparse lower hemispheric peripapillary atrophy. Furthermore, the presence of isolated patchy hypofluorescence in the left eye, which eluded detection during the fundus examination, suggests that atrophy in PPHCD may not consistently expand in a sequential manner.

Swept-source OCT imaging corroborated significant choroidal thinning and the absence of outer photoreceptor segments and RPE within the hypofluorescent area observed in FAF. This alignment between FAF and OCT findings underscores the link between hypofluorescence and retinal structural changes [11]. Additionally, OCTA revealed a notable choriocapillaris reduction, while the superficial and deep retinal capillary plexus appeared unaffected. This observation suggests that PPHCD primarily impacts the choriocapillaris, followed by subsequent retinal atrophy.

## Conclusions

The utilization of ultra-widefield FAF and OCTA may aid in the diagnosis and evaluation of PPHCD, especially in cases where the fundus exhibits a tigroid appearance. Long-term follow-up and further investigation of patients using these devices may provide insights into the progression of the disease.
